# Association of Genes in the High-Density Lipoprotein Metabolic Pathway with Polypoidal Choroidal Vasculopathy in Asian Population: A Systematic Review and Meta-Analysis

**DOI:** 10.1155/2018/9538671

**Published:** 2018-06-06

**Authors:** Ming-zhen Yuan, Ruo-an Han, Chen-xi Zhang, You-xin Chen

**Affiliations:** Department of Ophthalmology, Peking Union Medical College Hospital, Chinese Academy of Medical Sciences and Peking Union Medical College, Beijing, China

## Abstract

**Purpose:**

To assess the association of genes in the high-density lipoprotein metabolic pathway (HDLMP) with polypoidal choroidal vasculopathy (PCV) and the genetic difference in the HDLMP between PCV and age-related macular degeneration (AMD).

**Methods:**

We performed a literature search in EMBASE, PubMed, and Web of Science for genetic studies on 7 single nucleotide polymorphisms (SNPs) from 5 genes in the HDLMP including cholesteryl ester transfer protein (CETP), hepatic lipase (LIPC), lipoprotein lipase (LPL), ATP-binding cassette transporter A1 (ABCA1), and ATP-binding cassette transporter G1 (ABCG1) in PCV. All studies were published before September 30, 2017, without language restriction. Pooled odds ratios (ORs) and 95% confidence intervals (CIs) of each polymorphism were estimated. We also compared the association profiles between PCV and AMD and performed a sensitivity analysis.

**Results:**

Our result is based on 43 articles. After excluding duplicates and articles without complete information, 7 studies were applicable to meta-analysis. 7 polymorphisms were meta-analyzed: CETP rs2303790/rs3764261, LIPC rs10468017/rs493258, LPL rs12678919, ABCA1 rs1883025, and ABCG1 rs57137919. We found that in Asian population, CETP rs3764261 (T allele; OR = 1.46; 95% CI: 1.28–1.665, *P* < 0.01), CETP rs2303790 (G allele; OR = 1.57; 95% CI: 1.258–1.96, *P* < 0.01), and ABCG1 rs57137919 (A allele; OR = 1.168; 95% CI: 1.016–1.343, *P* < 0.01) were significantly associated with PCV, and ABCG1 rs57137919 (A allele; OR = 1.208, 95% CI: 1.035–1.411, *P* < 0.01) has different effects in PCV and AMD. The other 4 polymorphisms in LIPC/LPL/ABCA1 had no significant association with PCV (*P* > 0.05). The sensitivity analysis validated the significance of our analysis.

**Conclusions:**

Our study revealed 7 polymorphisms in 5 genes. Among them, CETP (rs3764261/rs2303790) and ABCG1 (rs57137919) were the major susceptibility genes for PCV in Asian population and ABCG1 (rs57137919) showed allelic diversity between PCV and AMD. Since the size for PCV and AMD was small, we need to study these genes genotyping in larger samples.

## 1. Introduction

Polypoidal choroidal vasculopathy (PCV) is a choroidal vascular disease of first described in the early 1980s as polypoidal subretinal vascular lesions associated with serous or hemorrhagic detachment of the retinal pigment epithelium (RPE) [1]. Later, PCV is regarded as a particular type of choroidal neovascularization (CNV) characterized by the distinct presence of polypoidal vascular lesions and a branching vascular network. PCV can be clearly demonstrated and diagnosed by indocyanine green angiography [2]. From a clinical perspective, PVC is considered as a subtype of AMD because of some similarities like neovascularization, subretinal hemorrhage and fluid, pigment epithelial detachment (PED), vision loss owing to bleeding, leakage, scar formation, and other similarities in phenotypic features [3–6]. Genetically, PCV and AMD also have the common susceptible genes, such as high temperature required factor A1 gene (HTRA1) and the complementary factor H gene (CFH) [7]. However, many controversial studies demonstrate that PCV should be classified as a distinct disease entity of AMD for their different epidemiological, clinical characteristics, natural history, and treatment outcomes [8–11]. Moreover, recent researches in the field of genetics suggest that PCV may not be as closely related to AMD, such as its differential risk to a mutation in FGD6, viralicidic activity 2-like (SKIV2L), complement component 3 (C3), elastin (ELN), and apolipoprotein E (APOE) [12–16]. These literatures make for a question whether PCV is a subtype of AMD or a distant disease from AMD.

Many studies have indicated lipid deposition in Bruch's membrane and soft drusen, and the amount of lipid was lower in the peripheral area than in the macula of human eyes [17]. A number of population-based studies revealed the association between drusen and the AMD, and drusen are regarded as one of the determinant factors of both early and late AMD [18, 19]. In addition, some studies indicated that the prevalence of drusen under RPE was lower in PCV than in AMD [20, 21], which pointed out that absence of drusen may be one of the important criteria to diagnose PCV. However, some clinical studies insisted that drusen were frequently seen in PCV [22, 23], and several studies reported that drusen were observed in 20% to 27% of unaffected [21, 24]. Therefore, whether drusen plays a functional role in the occurrence and development is still up for debate. As we know, lipids stand for over 40% of the drusen volume [25]; therefore, many academics studied the vital function of lipids in the pathogenesis of PCV and AMD. Due to the different ethnic groups and lifestyles of individuals, the strength of such relevances is widely variable [26–28]; therefore, many studies investigated the effect between gene variations in the HDLMP and risk factors on PCV and AMD. Genetic studies in the HDLMP with PCV and AMD have identified susceptibility single nucleotide polymorphisms (SNPs) in multiple genes, including rs3764261/rs2303790 in CETP, rs493258/rs10468017 in LIPC, rs12678919 in LPL, rs1883025 in ABCA1, and rs57137919 in ABCG1.

Thus far, some studies have studied the impact of lipid metabolism-related and systemic lipoprotein genes in PCV. Here, in order to give the comprehensive analysis of effects and solve the controversies, we conduct meta-analysis and report a systematic review by summing up all published articles of genetic associations in the HDLMP of PCV. This study (1) conducted an investigation of which genetic variants of the HDLMP are meaningfully associated with PCV and their effect sizes and (2) analyzed whether there were differences between genetic risks of the HDLMP in PCV and AMD.

## 2. Methods

### 2.1. Search Strategy

We searched EMBASE, PubMed, and Web of Science using the following MeSH terms and free words: (polypoidal choroidal vasculopathy or polypoidal choroidal vascular disease or polypoidal choroidal vascular diseases or PCV) and (cholesteryl ester transfer protein or CETP or hepatic lipase or LIPC or lipoprotein lipase or LPL or ATP-binding cassette transporter A1 or ABCA1 or ATP-binding cassette transporter G1 or ABCG1). All searched articles were published before September 30, 2017, without language restriction. We also screened the reference lists of all eligible studies, reviews, and meta-analyses to ensure that any relevant studies were not omitted. We also searched all reported genome-wide association studies of PCV including the supplementary materials to maximize the usable data. The detail of search strategy is revealed in Table S1.

### 2.2. Inclusion and Exclusion Criteria

We included those studies that satisfied the following criteria in the meta-analysis: (1) case-control studies, cohort studies, or population-based studies that evaluated the association of gene variants of CETP/LIPC/LPL/ABCA1/ABCG1 with PCV or its subtypes and (2) allele or genotype counts and/or frequencies being presented or able to be calculated from the data in the study. Case reports, conference reports, reviews, animal studies, and reports with insufficient information were excluded (Table S2).

### 2.3. Data Extraction and Quality Assessment

Two reviewers (Y. M. z. and Y. J. y.) independently reviewed and extracted data from studies on the association between CETP/LIPC/LPL/ABCA1/ABCG1 SNPs and PCV. If there were any differences between them, another two reviewers would help to resolve it (Z. C. x. and H. R. a.) after thorough discussion. The following information was extracted from each article: the name of first author, publication year, ethnicity of the study population, study design, genotyping method, sample size, demographics, allele and genotype distribution (Table 1), and the results of the Hardy–Weinberg equilibrium (HWE) test in controls (Table S3). We assessed the quality of individual studies using the Newcastle–Ottawa Scale [29]. Briefly, 9 quality indicators were used; if a study fulfilled 1 indicator, we assigned a “yes” under this item or a “no.” Thus, the quality score for each study might be between 0 and 9 (Table S4).

### 2.4. Statistical Analysis

We conducted meta-analysis for each polymorphism which had been reported in ≥2 studies or cohorts. The association was evaluated by different genetic models, including allelic, heterozygous, and homozygous models. For each study, the odds ratio (OR) with 95% confidence interval (95% CI) was calculated to evaluate the strength of association between the each SNP and PCV risk. Moreover, we used the *I* ^2^ value to quantify the proportion of the variability in effect estimates, which is due to heterogeneity rather than sampling error. The *I* ^2^ value was shown as of no (0–25%), low (25–50%), moderate (50–75%), or high heterogeneity (75–100%) [30]. The *I* ^2^ test was to assess heterogeneity among studies. The potential publication bias was assessed visually in a funnel plot of log (OR) against its standard error, and the degree of asymmetry was evaluated using Begg's test and Egger's test (*P* < 0.05 was considered to be statistically significant). We undertook the sensitivity analysis to examine the influence by removing the unreliable study [31]. The software STATA (version 12.0, StataCorp LP, College Station, TX) was used for the meta-regression analysis. A pooled *P* value of less than 0.05 was considered statistically significant.

## 3. Results

### 3.1. Eligibility and Characteristics of Included Studies

**Figure 1:**
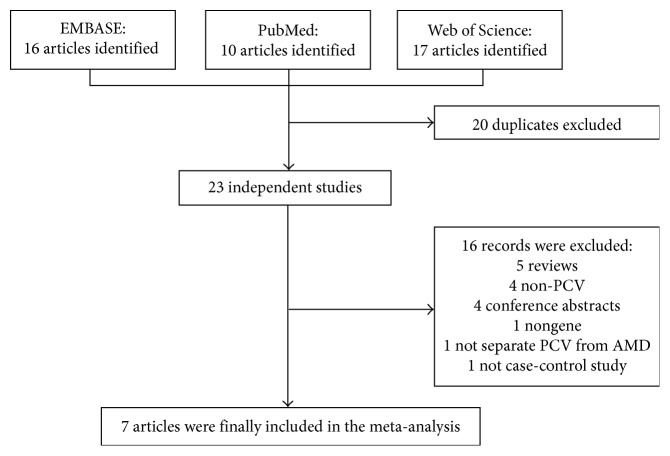
Flow diagram and results of literature review. The flow diagram describes the filtering process of related articles, including the number and reason of exclusion. PCV: polypoidal choroidal vasculopathy; AMD: age-related macular degeneration.

Figure 1 illustrates the study inclusion of this meta-analysis. A total of 43 articles published before September 30, 2017, were identified in the EMBASE, PubMed, and Web of Science databases. Of these, we excluded 20 articles because they were duplicates. For the remaining 23 studies, the full texts were retrieved. After reviewing the full texts, we excluded another 16 reports, among which 5 studies were reviews, 4 were not related to PCV, 4 were conference abstracts, 1 was not about the genetic studies, 1 did not separate PCV from AMD, and 1 was not the case-control study. Finally, 7 articles were eligible for the meta-analysis [32–38], involving 3342 PCV cases versus 8256 controls and 2761 PCV cases versus 2660 AMD cases. The main traits of the included studies are summed up in Table 1. Patients from each study received complete ophthalmic examinations, including fluorescein angiography and ICGA. Polypoidal choroidal vasculopathy was diagnosed on the basis of choroidal polypoidal lesions shown by ICGA. All studies adopted a case-control design. These studies were performed in various populations, including Chinese (6 studies), Japanese (3 studies), and Korean (1 study). In all studies, valid genotyping approaches were used, including polymerase chain reaction, TaqMan genotyping assay, and BeadChip.

### 3.2. Risk of Bias Assessment in Eligible Studies

As shown in Table S4, all eligible studies clearly described the diagnostic criteria for PCV and AMD. Patients with other macular diseases like central serous chorioretinopathy, myopic choroidal neovascularization, angioid streaks, presumed ocular histoplasmosis, or with CNV and PCV in the same or fellow eye were excluded. In all studies, comprehensive ophthalmic examinations were performed on the control subjects. One study used control subjects recruited from the community [32], and the others used hospital-recruited controls. Two studies were diverse-ethnic population study [37, 38]. One study did not provide the sex and age of cohorts. There was no ethnic difference between cases and controls. Confounding factors were matched between cases and controls in 7 studies. The scores for the quality assessment ranged from 5 to 7. All the studies informed HWE in controls.

### 3.3. Meta-Analysis of CETP/LIPC/LPL/ABCA1/ABCG1 Polymorphisms in PCV

**Figure 2:**
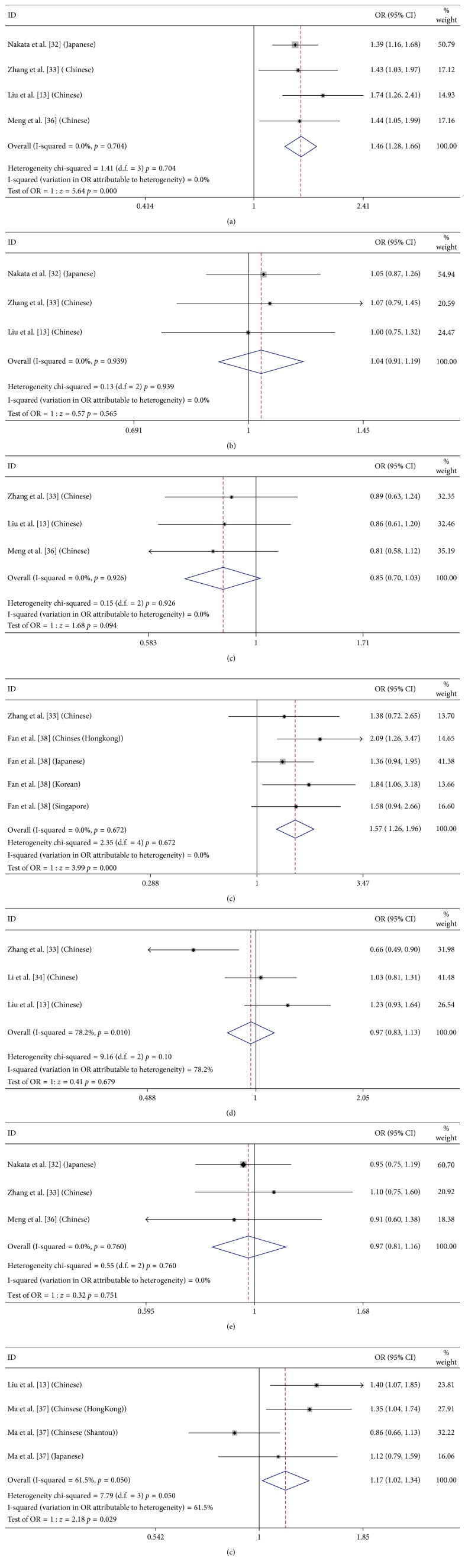
Forest plot of 7 SNPs in PCV in allelic model. The figure shows specific odds ratios (ORs) for study. The size of the box is proportional to the weight of the study. Horizontal lines represent 95% confidence intervals (CIs). A diamond is on behalf of the summary OR with its corresponding 95% CI. (a) CETP rs3764261 (T); (b) LIPC rs493258 (G); (c) LIPC rs10468017 (T); (d) CETP rs2303790 (G); (e) ABVA1 rs1883025 (T); (f) LPL rs12678919 (G); (g) ABCG1 rs57137919 (A).

Totally 17 SNPs had been studied in PCV in the literature (Figure 2). However, only 7 SNPs (CETP rs2303790/rs3764261, LIPC rs10468017/rs493258, LPL rs12678919, ABCA1 rs1883025, and ABCG1 rs57137919) in PCV were reported in more than one study and thus eligible for meta-analysis (details can be seen in Table S5). Summary of the allelic associations of these polymorphisms is shown in Table 2. The other 10 SNPs reported in only one report but performed no association with PCV [33, 36].

CETP rs3764261 is the most widely investigated SNP in PCV, with a number of 1355 cases and 1493 controls studied for the meta-analysis [32, 33, 35, 36]. The results showed statistically significant association between CETP rs3764261 and PCV in Asian population (Table 2). As for the allelic model, the odds ratio (OR) for the risk allele T was 1.46 (95% confidence interval (CI): 1.28–1.665, *P* < 0.01, *I* ^2^ = 0%). In the subgroup analysis by ethnicity, still significant association was detected in Chinese (OR = 1.528, 95% CI: 1.268–1.841, *P* < 0.01, *I* ^2^ = 0%). Also, CETP rs2303790 [33, 38] and ABCG1 rs57137919 [35, 37] showed significant associations with PCV in the allelic model. As for CETP rs2303790, the frequency of the G allele was significantly higher in PCV patients than in controls, conferring a 1.57-fold increased risk toward PCV (95% CI: 1.258–1.96, *P* < 0.01, *I* ^2^ = 0). As for ABCG1 rs57137919, the frequency of the A allele was significantly higher in PCV patients than in controls, conferring a 1.168-fold increased risk (95% CI: 1.016–1.343, *P*=0.029, *I* ^2^ = 61.5%). Through quality assessment and sensitivity analysis, we found the heterogeneity derived from the data of Shantou population [37]. After excluding the data of Shantou population, the result showed that the pooled allelic OR was significantly elevated (A allele; OR = 1.313, 95% CI: 1.113–1.548, *P* < 0.01, *I* ^2^ = 0%). Regarding the other 4 SNPs, LIPC rs10468017/rs493258, LPL rs12678919, and ABCA1 rs1883025, the pooled ORs were not statistically significant in PCV in the allelic (*P* > 0.05). As for ABCA1 rs1883025 (T allele; OR = 0.968, 95% CI: 0.828–1.131, *P*=0.679, *I* ^2^ = 78.2%), quality assessment and sensitivity analysis showed that the study of Zhang et al. was of higher risk of causing bias than the other cohorts [33]. Therefore, we excluded the study and also found that the pooled allelic OR was not significant (T allele; OR = 1.1, 95% CI: 0.926–1.335, *P*=0.257, *I* ^2^ = 0%).

### 3.4. Meta-Analysis of CETP/LIPC/LPL/ABCA1/ABCG1 Polymorphisms Compared between PCV and AMD

**Figure 3:**
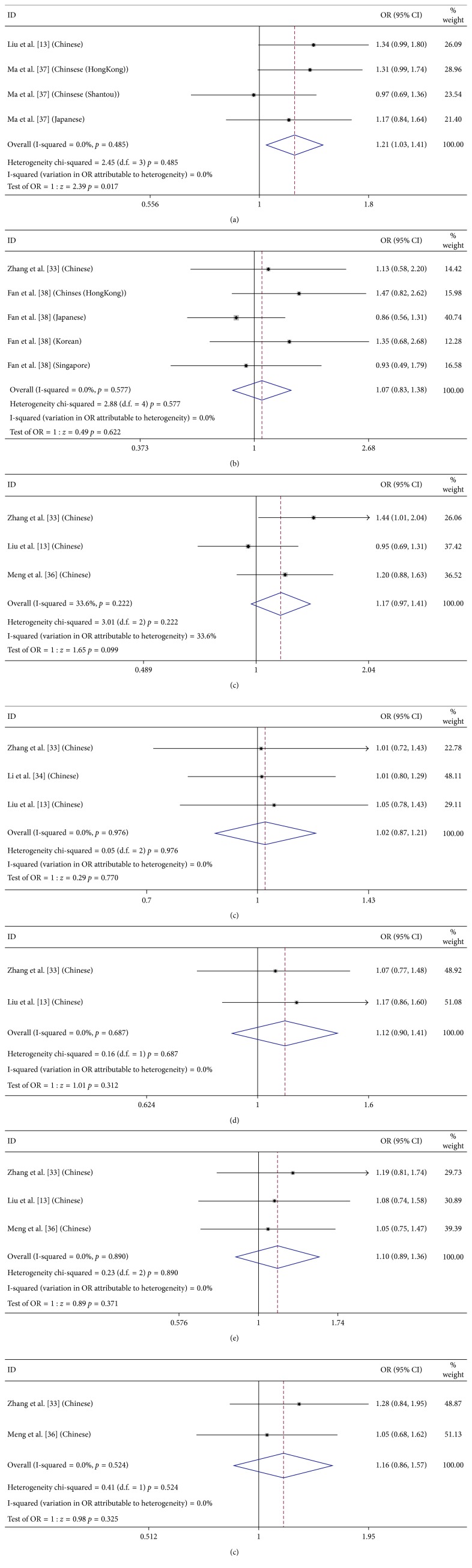
Forest plot of SNPs compared between PCV and AMD in allelic model. The figure shows specific odds ratios (ORs) for study. The size of the box is proportional to the weight of the study. Horizontal lines represent 95% confidence intervals (CIs). A diamond is on behalf of the summary OR with its corresponding 95% CI. (a) ABCG1 rs57137919 (A); (b) CETP rs2303790 (G); (c) CETP rs3764261 (T); (d) ABCA1 rs1883025 (T); (e) LIPC rs493258 (G); (f) LIPC rs10468017 (T); (g) LPL rs12678919 (G).

We identified 6 studies in which both PCV and AMD were assessed for associations with a total of 7 SNPs in 5 genes (i.e., CETP rs2303790/rs3764261, LIPC rs10468017/rs493258, LPL rs12678919, ABCA1 rs1883025, and ABCG1 rs57137919) (Table 3 and Figure 3). Only 1 SNP (ABCG1 rs57137919) showed significant difference between PCV and AMD (A allele; OR = 1.208, 95% CI: 1.035–1.411, *P*=0.017, *I* ^2^ = 0%) [35, 37]. The other 6 SNPs, namely, CETP rs2303790/rs3764261, LIPC rs10468017/rs493258, LPL rs12678919, and ABCA1 rs1883025, were evaluated in 2 to 3 cohorts and showed no significant differences between PCV and AMD (*P* > 0.05). As for CETP rs3764261 (T allele; OR = 1.17, 95% CI: 0.971–1.409, *P*=0.178, *I* ^2^ = 33.6%), quality assessment and sensitivity analysis showed that the study of Liu et al. was of higher risk of causing bias than the other cohorts [35]. Therefore, we ruled out the cohort, and then the pooled allelic OR of the result was significant (T allele; OR = 1.301, 95% CI: 1.033–1.638, *P*=0.025, *I* ^2^ = 0%) [33, 36].

### 3.5. Publication Bias Analysis

In theory, due to the limited number of available studies, it is not suitable for publication bias analysis. But in order to make this meta-analysis more powerful and more creditable, we used funnel plots and Begg's/Egger's test to detect publication bias. Begg's test and Egger's test suggested an absence of publication bias in the all SNPs (*P* > 0.05) (Tables 2 and 3). The shape of the funnel plots did not reveal any evidence of obvious asymmetry (Figures S1 and S2).

## 4. Discussion

In the systematic review and meta-analysis, we have summarized the association profiles of genes in the HDLMP in PCV and assessed the genetic difference in the HDLMP between PCV and AMD for the first time (i.e., CETP, LIPC, LPL, ABCA1, and ABCG1). We found significant association between reported CETP rs2303790/rs3764261, ABCG1 rs57137919, and PCV. Also, we identified ABCG1 rs57137919 showing significant differences between PCV and AMD. In contrast, LIPC rs10468017/rs493258, LPL rs12678919, and ABCA1 rs1883025 were not statistically significant in PCV and reported SNPs in 4 genes in the HDLMP (i.e., CETP, LIPC, LPL, and ABCA1) showed no significant differences between PCV and AMD.

CETP can make oxidized lipids transfer from the outer segments of the photoreceptors or other membranes to HDL-like lipoprotein particles. The particles are internalized by RPE and excreted back into the circulation via ABCG1 transporters through Bruch's membrane [39]. ABCG1 was relevant to an increased macrophage apoptosis, which may be due to the accumulation of oxysterol in macrophages caused by decreased ABCG1-mediated cholesterol efflux [40]. ABCA1 was expressed in the retina and retinal pigment epithelium [41], and ABCA1 can form nascent HDL by mediating the efflux of cholesterol and phospholipids to lipid-poor apolipoproteins [42, 43]. Also, several studies have demonstrated ABCA1 was significantly related to the progression of drusen, but the association between large drusen and geographic atrophy/neovascular was not significant [44]. Besides, LIPC gene is a critical enzyme in HDL metabolism which has the function of encoding hepatic triglyceride lipase and catalyzing the hydrolysis of phospholipids, monoglycerides, diglycerides, triglycerides, and acylCoA thioesters [45, 46]. LPL gene encodes LPL which can play an important role in HDL metabolism. LPL can not only facilitate triglyceride hydrolysis but also serve as a ligand/bridging factor for receptor-mediated lipoprotein uptake [47]. Besides, lipoproteins derived from plasma have been proved to be the crucial upstream source of fatty acids within Bruch's membrane and supply an energy source to the retina [48–50] as well as perform significant roles in the transportation of vitamin C, vitamin E, lutein, and zeaxanthin for use by photoreceptors [51, 52]. Therefore, dysfunction of CETP, LIPC, LPL, ABCA1, and ABCG1 may cause accumulation of oxidized lipids in the retina, and the unreasonable products could induce inflammation and vascular anomaly, which play a crucial role in the development of PCV and AMD via lipid metabolism [53, 54].

In the studies (including 3 in Chinese [33, 35, 36] and 1 in Japanese [32]), we found that CETP rs3764261 was to be associated with PCV in Asian population with an odds ratio of 1.46 (95% CI: 1.28–1.665, *P*=0, *I* ^2^ = 0%) for the T allele. Apart from CETP rs3764261, we also found that CETP rs2303790 was associated with PCV in Asian population [33, 38] (G allele; OR = 1.57, 95% CI: 1.258–1.96, *P* < 0.01, *I* ^2^ = 0). Therefore, CETP rs3764261/rs2303790 provided an increased risk for PCV in Asian population. Through the analysis of the studies in which both PCV and AMD were assessed for associations with CETP, we found CETP rs2303790 showed no significant difference between PCV and AMD. However, as for CETP rs3764261, two studies dedicated that CETP rs3764261 was significantly associated with an increased risk for PCV, but no association was found with AMD(T allele; OR = 1.301, 95% CI: 1.033–1.638, *P*=0.025, *I* ^2^ = 0%) [33, 40], but one study showed that CETP rs3764261 is a susceptibility gene for PCV and AMD [35]. According to the article of GWAS and meta-analysis published by Cheng et al., the minor allele at CETP rs3764261 variant was proved to be a risk factor to the development of AMD in Asian population [55]. However, several recent studies indicated that CETP rs3764261 was related to a decreased risk of AMD in Chinese population and Lithuanian population [56, 57]. Therefore, it is still disputed whether CETP rs3764261 has different effects in PCV and AMD, and we need further studies in which both PCV and AMD are assessed for associations with CETP to confirm it.

From the studies [35, 37], for ABCG1 rs57137919, the frequency of the A allele in PCV patients was significantly higher than in controls (OR = 1.168, 95% CI: 1.016–1.343, *P*=0.029, *I* ^2^ = 61.5%). Interestingly, we found the result had the heterogeneity. Through quality assessment and sensitivity analysis, we found the heterogeneity derived from the data of Shantou population. After reading the related articles, we found all the studies used TaqMan genotyping assays, as well as the same inclusion criteria and exclusion criteria. Therefore, we speculate that there may be racial differences between Shantou population and others, but we need further studies to prove the view and find the causes of heterogeneity. Also, we found the significant difference between PCV and AMD in ABCG1 rs57137919 (G allele; OR = 1.208, 95% CI: 1.035–1.411, *P*=0.017, *I* ^2^ = 0%). In one of the studies, Li et al. studied the relevance of ABCG1 rs57137919 to PCV and AMD in Hong Kong, Shantou, and Osaka study subjects, and the results showed that the association of ABCG1 rs57137919 with PCV was significant in the Hong Kong cohort, but not in the Shantou or the Osaka cohort. Also, they indicated that the association of ABCG1 rs57137919 with AMD was not significant in the Hong Kong, Shantou, and Osaka cohorts. In another study, Liu et al. provided putative evidence of a role of ABCG1 rs57137919 in the vascularized complication of PCV. Because there were only two studies about relevance of ABCG1 rs57137919 to PCV and AMD, we need further replication studies in other ethnic populations to confirm the role of ABCG1 in PCV and AMD.

From our meta-analysis, we found other 4 SNPs (LIPC rs10468017/rs493258, LPL rs12678919, and ABCA1 rs1883025) were not statistically significant in PCV and AMD. Besides, the associations of eleven SNPs in CETP/LIPC/ABCA1/ABCG1 were reported in only 1 study [33, 36–38] (details can be seen in Table S5). Among these SNPs, Zhang et al. discovered that the rs5882 variant in CETP was significantly associated with PCV (G allele; *P*=0.73*E*−04), but not with AMD (G allele; *P*=0.297), and suggested the need to find biological clues about the different underlying HDL pathways by separating PCV from AMD so as to explore the pathogenesis of PCV and AMD [33].

Another recent study reported that LIPC rs1532085 conferred an increased risk for PCV (A allele; *P*=0.0094), but not AMD (A allele; *P*=0.0938). Also, this study found hyperlipidemia is a risk factor for PCV [36]. Recently, Li et al. have newly identified ABCG1 rs225396 to be associated with PCV (T allele; *P*=0.026) and AMD (T allele; *P*=0.048) in Chinese and Japanese subjects, which pointed out ABCG1 as a new susceptibility gene for PCV and AMD [37]. In 2017, Qiao et al. reported that the strongest PCV-associated SNP, CETP rs183130 (T allele; *P*=3.07*E*−07), was in high LD with the currently studied SNP rs3764261 in Europeans, and similar association patterns were shown in AMD at CETP rs183130 (T allele; *P*=4.31*E*−05). Also, they found that the most significant association signals recognized in Europeans were at rs5817082, rs1864163, and rs17231506 in CETP, but only rs17232506 showed significant association with PCV and AMD [38]. However, all the results above were reported in only 1 study, and they were challenged by pretty small sample sizes, so we need more researches with independent cohorts to verify these association findings.

In conclusion, our systematic review and meta-analysis has provided an overview of the association profiles of genes in the HDLMP in PCV for the first time and assessed the genetic difference in the HDLMP between PCV and AMD. The results suggest that CETP (rs3764261/rs2303790) and ABCG1 (rs57137919) are the major susceptibility genes for PCV in the Asian population, and ABCG1 (rs57137919) has different effects in PCV and AMD in the Asian population. However, due to the small pooled sample size for PCV and AMD, further studies of these genes in larger samples are warranted to confirm the association of gene variations in the HDLMP with PCV in other populations such as Caucasian and Australian. Moreover, further studies should focus on the genotype-phenotype correlations and the relevance of genotype to therapy in PCV, which may provide us the clues about the pathogenesis of PCV.

## Figures and Tables

**Table 1:** Characteristics of the included studies in the meta-analysis.

First author and reference	Year	Ethnicity	Study design	Genotyping method	HWE reported	PCV			AMD			Control			Gene/loci investigated
						*N*	Male ratio	Mean age ± SD (yrs)	*N*	Male ratio	Mean age ± SD (yrs)	*N*	Male ratio	Mean age ± SD (yrs)	
Nakata et al. [32]	2013	Japanese	1	TaqMan and Beadchip	Yes	581	0.73	72.59 ± 8.13	—	—	—	793	0.41	65.99 ± 4.33	CETP, LIPC, LPL
Zhang et al. [33]	2013	Chinese	1	PCR	Yes	250	0.66	65 ± 8.6	157	0.64	67 ± 9.21	204	0.61	69 ± 9	LIPC, ABCA1, CETP, LPL,
Li et al. [34]	2014	Chinese	1	PCR	Yes	298	0.62	66.8 ± 9.7	300	0.63	69.4 ± 8.9	296	0.48	65.1 ± 9.5	ABCA1
Liu et al. [13]	2014	Chinese	1	PCR	Yes	233	0.7	68.5 ± 5.9	200	0.55	75.3 ± 7.7	275	0.44	74.3 ± 7.6	ABCA1, LIPC, CETP, ABCG1
Meng et al. [36]	2015	Chinese	1	PCR	Yes	291	0.79	66.6 ± 9.6	230	0.63	69.3 ± 8.8	221	0.48	67.2 ± 9.6	CETP, LIPC, LPL
Li et al. [37]	2016	Chinese (Hong Kong)	1	TaqMan and PCR	Yes	236	0.69	68.5 ± 9	235	0.55	75.3 ± 7.6	365	0.42	74.4 ± 7.7	ABCG1
	2016	Chinese (Shantou)	1	TaqMan and PCR	Yes	187	0.72	63.1 ± 10.5	189	0.69	67.3 ± 10.1	670	0.43	73.8 ± 6.8	ABCG1
	2016	Japanese (Osaka)	1	TaqMan and PCR	Yes	204	0.77	72.2 ± 8.0	192	0.67	74.3 ± 7.3	157	0.33	47.9 ± 15.1	ABCG1
Qiao et al. [38]	2017	Chinese (Hong Kong)	1	Beadchip	Yes	156	—	—	310	—	—	1006	—	—	CETP

The characteristics of the eligible studies are shown. PCV: polypoidal choroidal vasculopathy; AMD: age-related macular degeneration; HWE: Hardy–Weinberg equilibrium.

**Table 2:** Meta-analysis of CETP/LIPC/LPL/ABCA1/ABCG1 polymorphisms in PCV.

Region	Gene	Polymorphism	Ethnicity	Associated versus reference allele	Number of cohorts	Sample size (case/control)	OR (95% CI)	*Z* score	*P* value	*I* ^2^ (%)	*P* value of Begg's test	*P* value of Egger's test
16q21	*CETP*	rs3764261	Asia	T versus G	4	1355/1493	1.46 (1.28–1.665)	5.64	0	0	0.308	0.415
			Chinese	T versus G	3	774/700	1.528 (1.268–1.841)	4.46	0	0	>0.999	0.071
9q31	*ABCA1*	rs1883025	Chinese	T versus C	3	781/775	0.968 (0.828–1.131)	0.41	0.679	78.2	>0.999	0.745
9q31	*LIPC*	rs493258	Asia	G versus A	3	1064/1272	1.041 (0.907–1.195)	0.57	0.565	0	>0.999	0.867
15q22	*LIPC*	rs10468017	Chinese	T versus C	3	774/700	0.849 (0.7–1.028)	1.68	0.094	0	>0.999	0.465
15q22	*LPL*	rs12678919	Asia	G versus A	3	1122/1218	0.972 (0.814–1.161)	0.32	0.751	0	>0.999	0.799
21q22	*ABCG1*	rs57137919	Asia	A versus G	4	860/1467	1.168 (1.016–1.343)	2.18	0.029	61.5	>0.999	0.808
16q21	CETP	rs2303790	Asia	G versus A	5	1312/5479	1.57 (1.258–1.96)	3.99	0	0	>0.999	0.527

Summary of the allelic associations of CETP/LIPC/LPL/ABCA1/ABCG1 polymorphisms in PCV. PCV: polypoidal choroidal vasculopathy; AMD: age-related macular degeneration; OR: odds ratio; CI: confidence intervals.

**Table 3:** Meta-analysis of CETP/LIPC/LPL/ABCA1/ABCG1 polymorphisms compared between PCV and AMD.

Region	Gene	Polymorphism	Ethnicity	Associated versus reference allele	Number of cohorts	Sample size (PCV/AMD)	OR (95% CI)	*Z* score	*P* value	*I* ^2^ (%)	*P* value of Begg's test	*P* value of Egger's test
16q21	*CETP*	rs3764261	Chinese	T versus G	3	774/587	1.17 (0.971–1.409)	1.65	0.178	33.6	>0.999	0.572
9q31	*ABCA1*	rs1883025	Chinese	T versus C	3	781/775	1.025 (0.869–1.208)	0.29	0.77	0	>0.999	0.832
9q31	*LIPC*	rs493258	Chinese	G versus A	2	483/357	1.123 (0.897–1.407)	1.01	0.312	0	>0.999	—
15q22	*LIPC*	rs10468017	Chinese	T versus C	3	774/587	1.101 (0.892–1.359)	0.89	0.371	0	0.296	0.483
15q22	*LPL*	rs12678919	Asia	G versus A	2	541/387	1.163 (0.861–1.572)	0.98	0.325	0	>0.999	—
21q22	*ABCG1*	rs57137919	Asia	A versus G	4	860/816	1.208 (1.035–1.411)	2.39	0.017	0	0.734	0.156
16q21	CETP	rs2303790	Asia	G versus A	5	1312/1314	1.067 (0.825–1.378)	0.49	0.622	0	0.462	0.299

Summary of the genetic difference in CETP/LIPC/LPL/ABCA1/ABCG1 polymorphisms between PCV and AMD is shown. PCV: polypoidal choroidal vasculopathy; AMD: age-related macular degeneration; OR: odds ratio; CI: confidence interval.

## Supplementary Materials

Supplementary Materials Table S1: the search strategy applied in all databases. Table S2: lists of included/excluded studies with reasons. Table S3: Hardy–Weinberg equilibrium of polymorphisms in control subjects. Table S4: quality assessment of each study based on the Newcastle–Ottawa Scale. Table S5: the number of studies for CETP/LIPC/LPL/ABCA1/ABCG1 polymorphisms in PCV. Figure S1: funnel plot of 7 SNPs in PCV in the allelic model. Figure S2: funnel plot of 7 SNPs compared between PCV and AMD in the allelic model.
