# Supplementary material for: Association of Genes in the High-Density Lipoprotein Metabolic Pathway with Polypoidal Choroidal Vasculopathy in Asian Population: A Systematic Review and Meta-Analysis
Source: J Ophthalmol. 2018 Jun 6;2018:9538671. doi: 10.1155/2018/9538671 (PMC6011074; doi:10.1155/2018/9538671)
Supplement: Supplementary Materials — Table S1: the search strategy applied in all databases. Table S2: lists of included/excluded studies with reasons. Table S3: Hardy–Weinberg equilibrium of polymorphisms in control subjects. Table S4: quality assessment of each study based on the Newcastle–Ottawa Scale. Table S5: the number of studies for CETP/LIPC/LPL/ABCA1/ABCG1 polymorphisms in PCV. Figure S1: funnel plot of 7 SNPs in PCV in the allelic model. Figure S2: funnel plot of 7 SNPs compared between PCV and AMD in the allelic model. [file 9538671.f1.docx]

**Appendix 1. Database search**

**Search strategy for EMBASE:**

1. Polypoidal choroidal vasculopathy
2. Polypoidal choroidal vascular disease
3. Polypoidal choroidal vascular diseases
4. PCV
5. cholesteryl ester transfer protein
6. CETP
7. hepatic lipase
8. LIPC
9. lipoprotein lipase
10. LPL
11. ATP-binding cassette transporter A1
12. ABCA1
13. ATP-binding cassette transporter G1
14. ABCG1
15. (1 OR 2 OR 3 OR 4)
16. (6 OR 7 OR 8 OR 9 OR 10 OR 11 OR 12 OR 13 OR 14)
17. 15 AND 16

**Search strategy for PubMed:**

(((((Polypoidal choroidal vasculopathy) OR Polypoidal choroidal vascular disease) OR Polypoidal choroidal vascular diseases) OR PCV)) AND ((((((((((cholesteryl ester transfer protein) OR hepatic lipase) OR lipoprotein lipase) OR ATP-binding cassette transporter A1) OR ATP-binding cassette transporter G1) OR CETP) OR LIPC) OR LPL) OR ABVA1) OR ABCG1)

**Search strategy for Web of Science:**

1. (Polypoidal choroidal vasculopathy OR Polypoidal choroidal vascular disease OR Polypoidal choroidal vascular diseases OR PCV)
2. (cholesteryl ester transfer protein OR CETP OR hepatic lipase OR LIPC OR lipoprotein lipase OR LPL OR ATP-binding cassette transporter A1 OR ABCA1 OR ATP-binding cassette transporter G1 OR ABCG1)
3. (1 AND 2)

**Appendix 2**

**Lists of included/excluded studies with reasons**

| **No.** | **Year** | **First Author** | **Journal** | **Title** | **Reason for**  **Inclusion/exclusion** |
| --- | --- | --- | --- | --- | --- |
| **Included**  **studies** |  |  |  |  |  |
| 1 | 2013 | Nakata I | Investigative Ophthalmology and Visual Science | Association between the cholesteryl ester transfer protein gene and polypoidal choroidal vasculopathy | Eligibility |
| 2 | 2013 | Zhang X | Experimental Eye Research | Different impact of high-density lipoprotein-related genetic variants on polypoidal choroidal vasculopathy and neovascular age-related macular degeneration in a Chinese Han population | Eligibility |
| 3 | 2014 | Li F | Ophthalmic Research | ABCA1 rs1883025 polymorphism shows no association with neovascular age-related macular degeneration or polypoidal choroidal vasculopathy in a Northern Chinese population | Eligibility |
| 4 | 2014 | Liu K | Ophthalmology | Genes in the high-density lipoprotein metabolic pathway in age-related macular degeneration and polypoidal choroidal vasculopathy | Eligibility |
| 5 | 2015 | Meng Q | PLoS One | Effect of High-Density Lipoprotein Metabolic Pathway Gene Variations and Risk Factors on Neovascular Age-Related Macular Degeneration and Polypoidal Choroidal Vasculopathy in China | Eligibility |
| 6 | 2016 | Ma L | Investigative Ophthalmology and Visual Science | Association of ABCG1 With Neovascular Age-Related Macular Degeneration and Polypoidal Choroidal Vasculopathy in Chinese and Japanese | Eligibility |
| 7 | 2017 | Fan Q | Journal of Human Genetics | Shared genetic variants for polypoidal choroidal Shared genetic variants for polypoidal choroidalvasculopathy and typical neovascular age-related macular degeneration in East Asians | Eligibility |
| **Excluded**  **studies** |  |  |  |  |  |
| 1 | 1992 | Yagi Y | Journal of Veterinary Medical Science | Plasma High-Density-Lipoprotein in Anemic Cattle Infected with Theileria-Sergenti | None-PCV |
| 2 | 2008 | Samadi S | Advances in Computer and Information Sciences and Engineering | Vehicle Detection Using a Multi-Agent Vision-Based System | None-PCV |
| 3 | 2012 | Hegerova L | American Journal of Gastroenterology | A rapidly progressive case of hemorrhagic pancreatitis in a patient with polycythemia vera, or not? | Conference abstracts |
| 4 | 2012 | Kortvely E | Retinal Degenerative Diseases | Common mechanisms for separate maculopathies? | Not case-control study |
| 5 | 2013 | Kuo J | Expert Review of Ophthalmology | Genetic risk, ethnic variations and pharmacogenetic biomarkers in AMD and polypoidal choroidal vasculopathy | Review |
| 6 | 2013 | Nowak-Sliwinska P | Progress in Retinal and Eye Research | Photodynamic therapy for polypoidal choroidal vasculopathy | None-gene |
| 7 | 2014 | Van L | Journal of Pathology | Mechanisms of age-related macular degeneration and therapeutic opportunities | Review |
| 8 | 2015 | Ma L | Investigative Ophthalmology & Visual Science | ABCG1 as a new susceptibility gene for age-related macular degeneration and polypoidal choroidal vasculopathy | Conference abstracts |
| 9 | 2015 | Ma L | Ophthalmology | Association of Genetic Variants with Polypoidal Choroidal Vasculopathy: A Systematic Review and Updated Meta-analysis | Review |
| 10 | 2015 | Ma L | Investigative Ophthalmology and Visual Science | As a new susceptibility gene for agerelated macular degeneration and polypoidal choroidal vasculopathy | Conference abstracts |
| 11 | 2016 | Wang Y | Graefes Archive for Clinical and Experimental Ophthalmology | ABCA1 rs1883025 polymorphism and risk of age-related macular degeneration | Review |
| 12 | 2016 | Pang C | Investigative Ophthalmology and Visual Science | ABCG1 as a new susceptibility gene for neovascular age-related macular degeneration and polypoidal choroidal vasculopathy in Chinese and Japanese | Conference abstracts |
| 13 | 2016 | Maguire M | Jama Ophthalmology | Single-Nucleotide Polymorphisms Associated With Age-Related Macular Degeneration and Lesion Phenotypes in the Comparison of Age-Related Macular Degeneration Treatments Trials | None-PCV |
| 14 | 2017 | Cheung C | Journal of Lipid Research | Plasma lipoprotein subfraction concentrations are associated with lipid metabolism and age-related macular degeneration | None-PCV |
| 15 | 2017 | Kim J | Current Ophthalmology Reports | Polypoidal Choroidal Vasculopathy | Review |
| 16 | 2016 | Momozawa Y | Human Molecular Genetics | Low-frequency coding variants in CETP and CFB are associated with susceptibility of exudative age-related macular degeneration in the Japanese population | Didn’t separate PCV from AMD |

**Appendix 3**

**Hardy-Weinberg equilibrium of polymorphisms in control subjects**

| **No.*** | **Polymorphism** | **Gene** | **Year** | **First Author** | **P value** |
| --- | --- | --- | --- | --- | --- |
| 1 | rs3764261 | *CETP* | 2013 | Nakata I | 0.9 |
| 1 | rs3764261 | *CETP* | 2013 | Zhang X | 0.87 |
| 1 | rs3764261 | *CETP* | 2014 | Liu K | 0.92 |
| 1 | rs3764261 | *CETP* | 2015 | Meng QY | 0.25 |
| 2 | rs1883025 | *ABCA1* | 2013 | Zhang X | 0.9 |
| 2 | rs1883025 | *ABCA1* | 2014 | Li F | 0.9 |
| 2 | rs1883025 | *ABCA1* | 2014 | Liu K | 0.96 |
| 3 | rs493258 | *LIPC* | 2013 | Nakata I | 0.73 |
| 3 | rs493258 | *LIPC* | 2013 | Zhang X | 0.94 |
| 3 | rs493258 | *LIPC* | 2013 | Liu K | 0.94 |
| 4 | rs10468017 | *LIPC* | 2013 | Zhang X | 0.86 |
| 4 | rs10468017 | *LIPC* | 2014 | Liu K | 0.92 |
| 4 | rs10468017 | *LIPC* | 2015 | Meng QY | 0.35 |
| 5 | rs12678919 | *LPL* | 2013 | Nakata I | 0.86 |
| 5 | rs12678919 | *LPL* | 2013 | Zhang X | 0.87 |
| 5 | rs12678919 | *LPL* | 2015 | Meng QY | 0.89 |
| 6 | rs57137919 | *ABCG1* | 2013 | Zhang X | ＞0.05^#^ |
| 6 | rs57137919 | *ABCG1* | 2016 | Li M | ＞0.05^#^ |
| 7 | rs2303790 | *CETP* | 2017 | Qiao F | ＞10^-6#^ |

* indicates that the same SNP reported in different studies was assigned a same number. ^#^ indicates that we didn’t find the concrete value of Hardy-Weinberg equilibrium of polymorphisms in control subjects, but HWE was one of exclusion criterias in all three studies.

Gene symbols: CETP = cholesteryl ester transfer protein- plasma; ABCA1 = ATP-binding cassette, sub-family A; LIPC = lipase hepatic; LPL = lipoprotein lipase; ABCG1 = ATP-binding cassette transporter G1.

**Appendix 4**

**Quality assessment of each study based on the Newcastle-Ottawa Scale**

| **First Author** | **Year** | **Quality assessment for individual study from Newcastle-Ottawa Scale** | | | | | | | | |
| --- | --- | --- | --- | --- | --- | --- | --- | --- | --- | --- |
|  |  | **1)** | **2)** | **3)** | **4)** | **5)** | **6)** | **7)** | **8)** | **9)** |
| Nakata I | 2013 | Yes | Yes | Yes | Yes | Yes | Yes | No | Yes | No |
| Zhang X | 2013 | Yes | Yes | No | Yes | Yes | Yes | No | Yes | No |
| Liu K | 2014 | Yes | Yes | No | Yes | Yes | Yes | No | Yes | No |
| Li F | 2014 | Yes | Yes | No | Yes | Yes | Yes | No | Yes | No |
| Meng QY | 2015 | Yes | Yes | No | Yes | Yes | Yes | No | Yes | No |
| Li Ma | 2016 | Yes | Yes | Yes | Yes | Yes | Yes | No | Yes | No |
| Qiao F | 2017 | Yes | Yes | No | Yes | Yes | No | No | Yes | No |

1) independent validation of PCV and/or AMD; 2) representative population for PCV and/or AMD; 3) community controls; 4) clear definition for controls; 5) no ethnic difference between cases and controls; 6) study controlled for confounding factors, e.g., age, gender, and/or smoking; 7) ascertainment of exposure by blinded interview or record; 8) same method of ascertainment for cases and controls; and 9) same non-response rate for cases and controls.

**Appendix 5**

**Characteristics of the included studies in the meta-analysis**

| **Gene** | **Number of studies** | | | |
| --- | --- | --- | --- | --- |
|  | **1** | **2** | **3** | **4** |
| CETP | rs5882 | rs2303790 |  | rs3764261 |
|  | rs173539 |  |  |  |
|  | rs183130 |  |  |  |
|  | rs5817082 |  |  |  |
|  | rs1864163 |  |  |  |
|  | rs17231506 |  |  |  |
|  |  |  |  |  |
| LIPC | rs493258 |  | rs10468017 |  |
|  | rs6078 |  | rs493258 |  |
|  | rs6083 |  |  |  |
|  | rs3829462 |  |  |  |
|  | rs1532085 |  |  |  |
|  |  |  |  |  |
| LPL |  |  | rs12678919 |  |
|  |  |  |  |  |
| ABCA1 | rs1883025 |  | rs1883025 |  |
|  | rs2066715 |  |  |  |
|  | rs2066718 |  |  |  |
|  |  |  |  |  |
| ABCG1 | rs225396 | rs57137919 |  |  |

**Appendix 6**

**Funnel plot of 7 SNPs in PCV in allelic model**

**
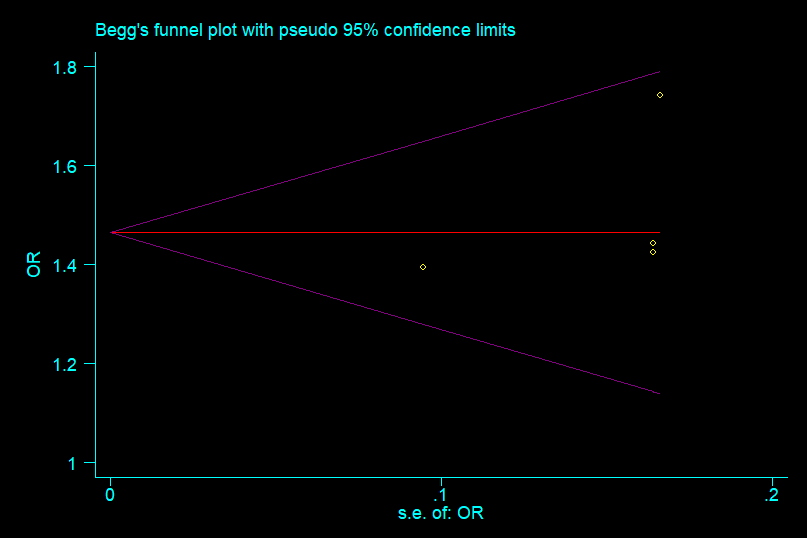
**

**The Begg funnel plot of CETP rs3764261 in Asian population.** The vertical axis represents OR and the horizontal axis means the standard error (SE) of OR. horizontal line and sloping lines in the funnel plot represent random effect summary OR and expected 95%CI for a given SE, respectively. Each circle area represents the contribution of each study to the pooled OR


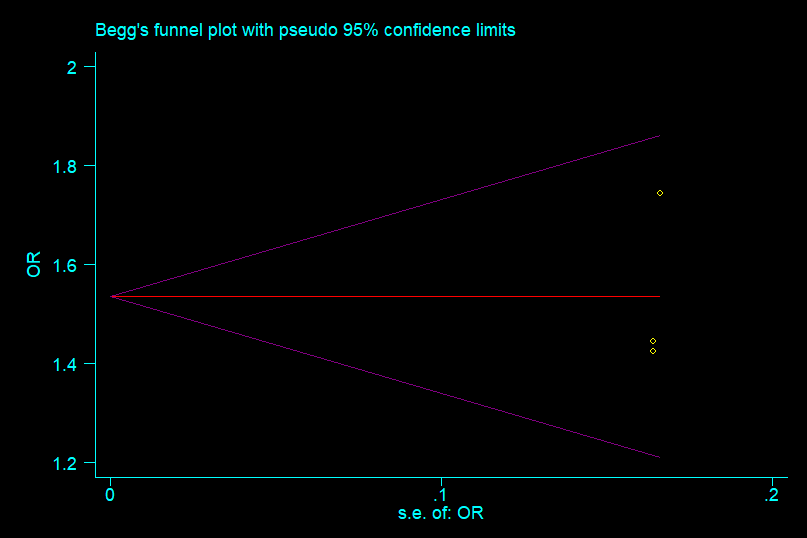


**The Begg funnel plot of CETP rs3764261 in Chinese population.** The vertical axis represents OR and the horizontal axis means the standard error (SE) of OR. Horizontal line and sloping lines in the funnel plot represent random effect summary OR and expected 95%CI for a given SE, respectively. Each circle area represents the contribution of each study to the pooled OR


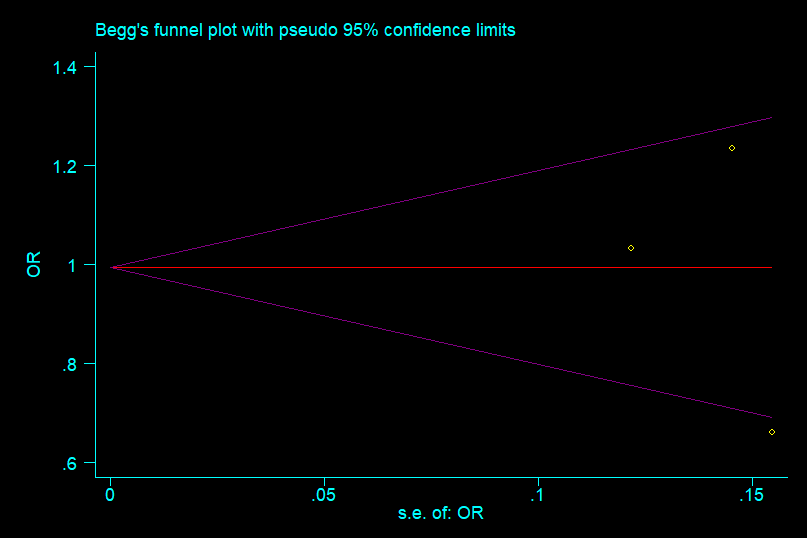


**The Begg funnel plot of ABCA1 rs1883025.** The vertical axis represents OR and the horizontal axis means the standard error (SE) of OR. Horizontal line and sloping lines in the funnel plot represent random effect summary OR and expected 95%CI for a given SE, respectively. Each circle area represents the contribution of each study to the pooled OR


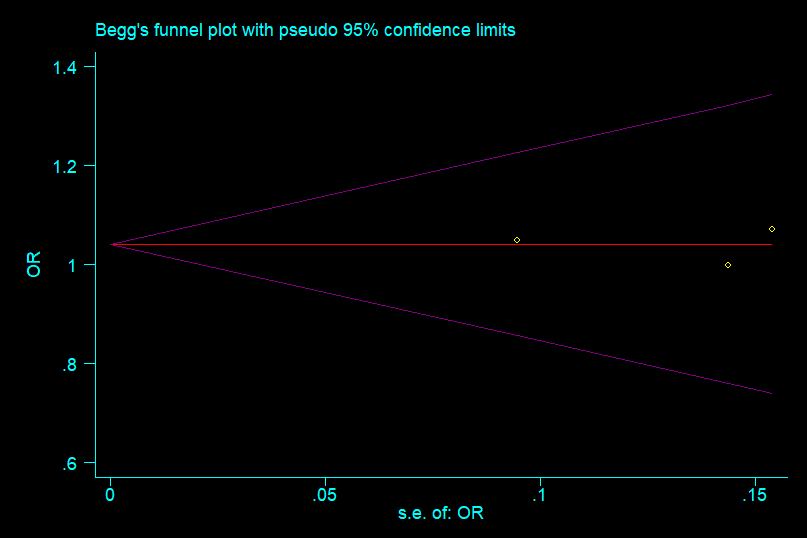


**The Begg funnel plot of LIPC rs493258.** The vertical axis represents OR and the horizontal axis means the standard error (SE) of OR. Horizontal line and sloping lines in the funnel plot represent random effect summary OR and expected 95%CI for a given SE, respectively. Each circle area represents the contribution of each study to the pooled OR


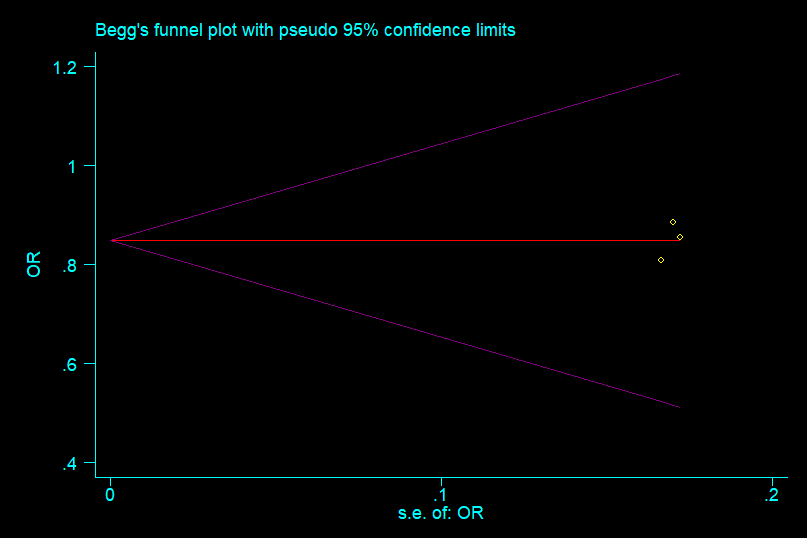


**The Begg funnel plot of LIPC rs10468017.** The vertical axis represents OR and the horizontal axis means the standard error (SE) of OR. Horizontal line and sloping lines in the funnel plot represent random effect summary OR and expected 95%CI for a given SE, respectively. Each circle area represents the contribution of each study to the pooled OR


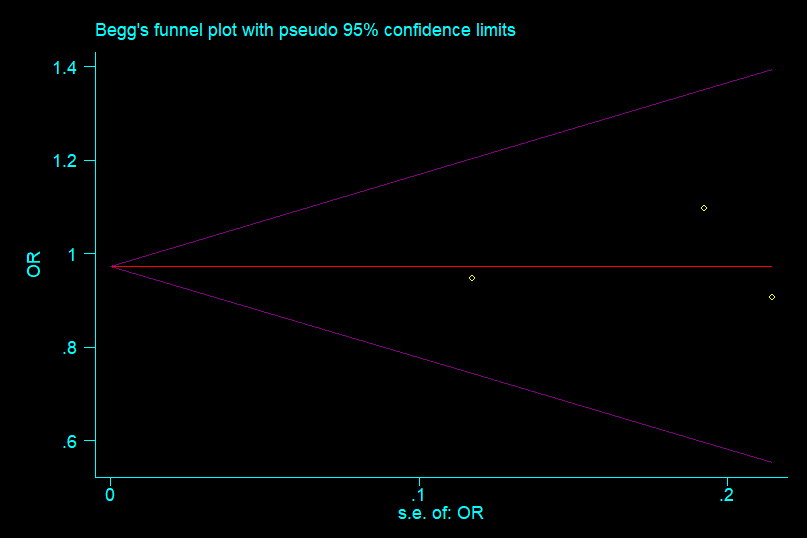


**The Begg funnel plot of LPL rs12678919.** The vertical axis represents OR and the horizontal axis means the standard error (SE) of OR. Horizontal line and sloping lines in the funnel plot represent random effect summary OR and expected 95%CI for a given SE, respectively. Each circle area represents the contribution of each study to the pooled OR


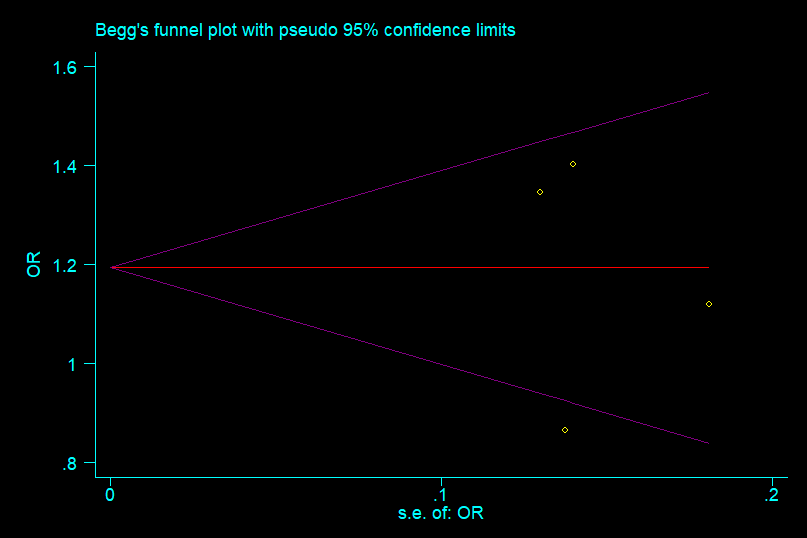


**The Begg funnel plot of ABCG1 rs57137919.** The vertical axis represents OR and the horizontal axis means the standard error (SE) of OR. Horizontal line and sloping lines in the funnel plot represent random effect summary OR and expected 95%CI for a given SE, respectively. Each circle area represents the contribution of each study to the pooled OR


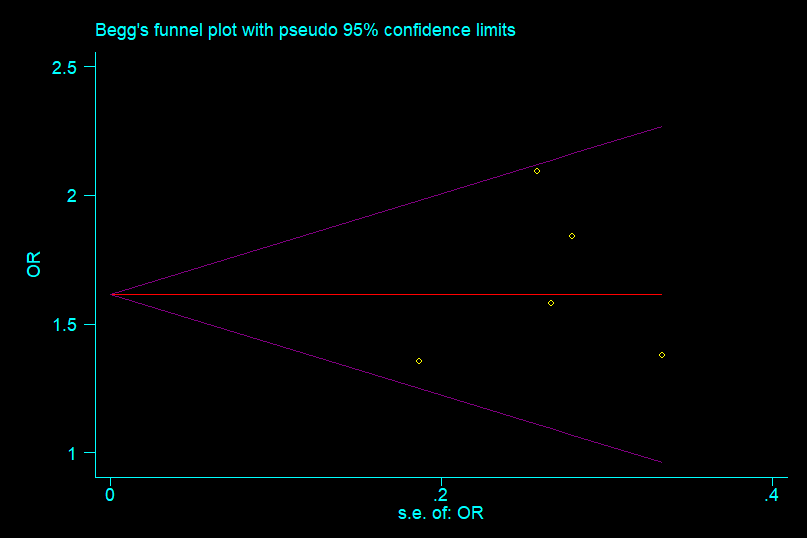


**The Begg funnel plot of CEPT rs2303790.** The vertical axis represents OR and the horizontal axis means the standard error (SE) of OR. Horizontal line and sloping lines in the funnel plot represent random effect summary OR and expected 95%CI for a given SE, respectively. Each circle area represents the contribution of each study to the pooled OR

**Appendix 7**

**Funnel plot of 7 SNPs in the compare between PCV and AMD in allelic mode**


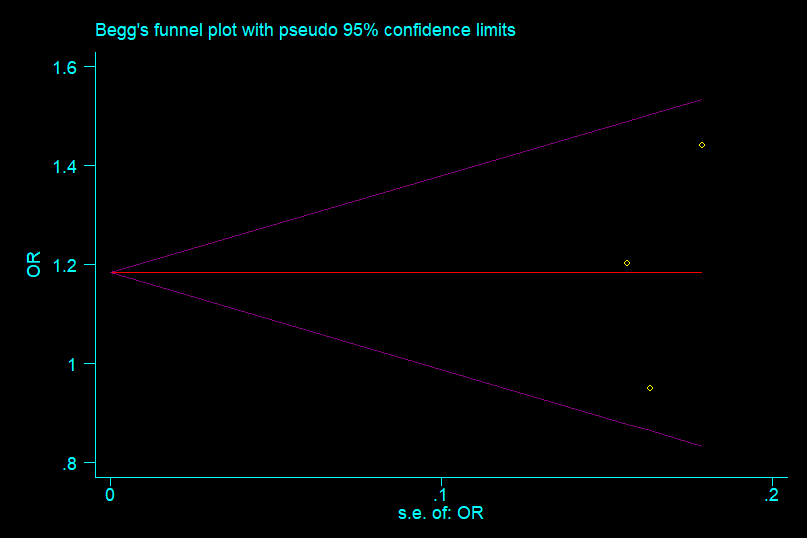


**The Begg funnel plot of CETP rs3764261.** The vertical axis represents OR and the horizontal axis means the standard error (SE) of OR. horizontal line and sloping lines in the funnel plot represent random effect summary OR and expected 95%CI for a given SE, respectively. Each circle area represents the contribution of each study to the pooled OR


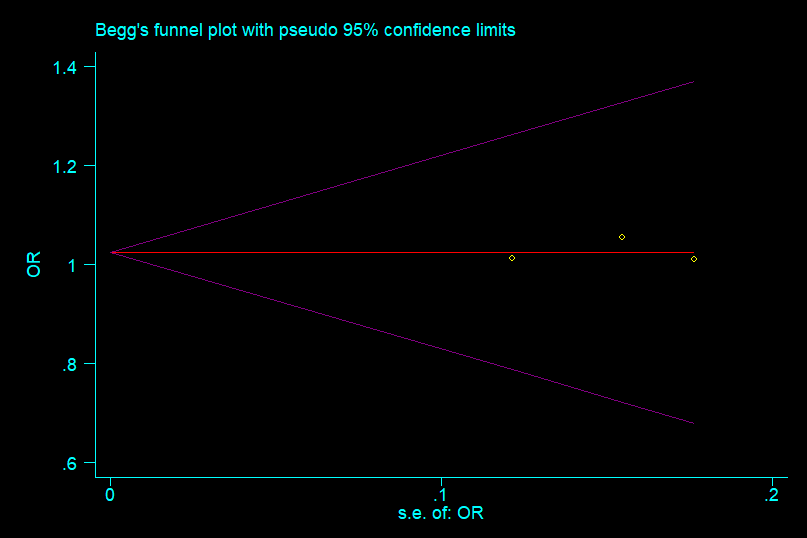


**The Begg funnel plot of ABCA1 rs1883025.** The vertical axis represents OR and the horizontal axis means the standard error (SE) of OR. Horizontal line and sloping lines in the funnel plot represent random effect summary OR and expected 95%CI for a given SE, respectively. Each circle area represents the contribution of each study to the pooled OR


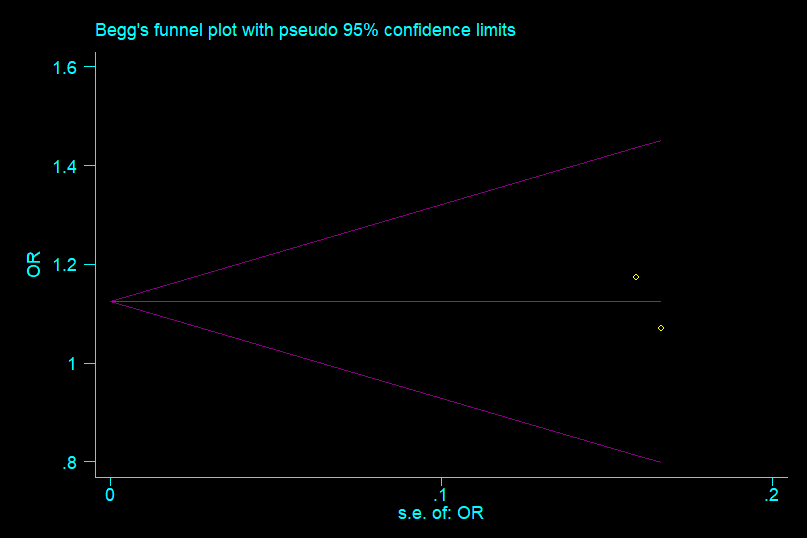


**The Begg funnel plot of LIPC rs493258.** The vertical axis represents OR and the horizontal axis means the standard error (SE) of OR. Horizontal line and sloping lines in the funnel plot represent random effect summary OR and expected 95%CI for a given SE, respectively. Each circle area represents the contribution of each study to the pooled OR


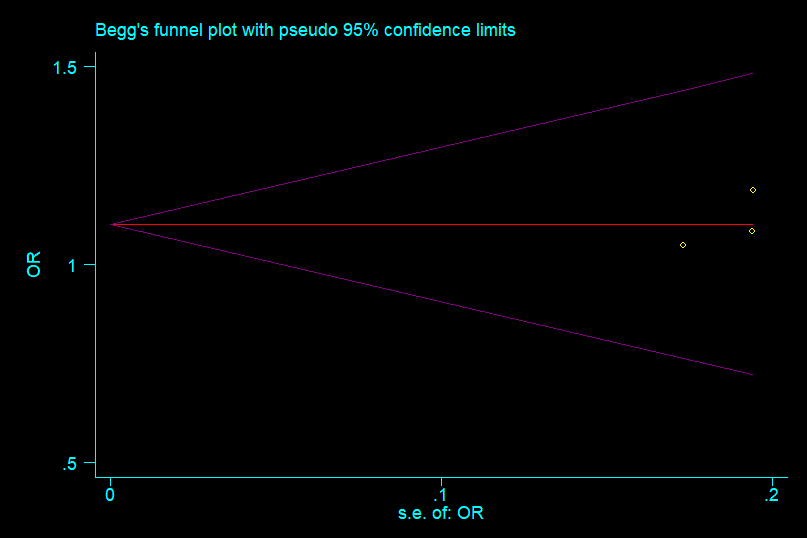


**The Begg funnel plot of LIPC rs10468017.** The vertical axis represents OR and the horizontal axis means the standard error (SE) of OR. Horizontal line and sloping lines in the funnel plot represent random effect summary OR and expected 95%CI for a given SE, respectively. Each circle area represents the contribution of each study to the pooled OR


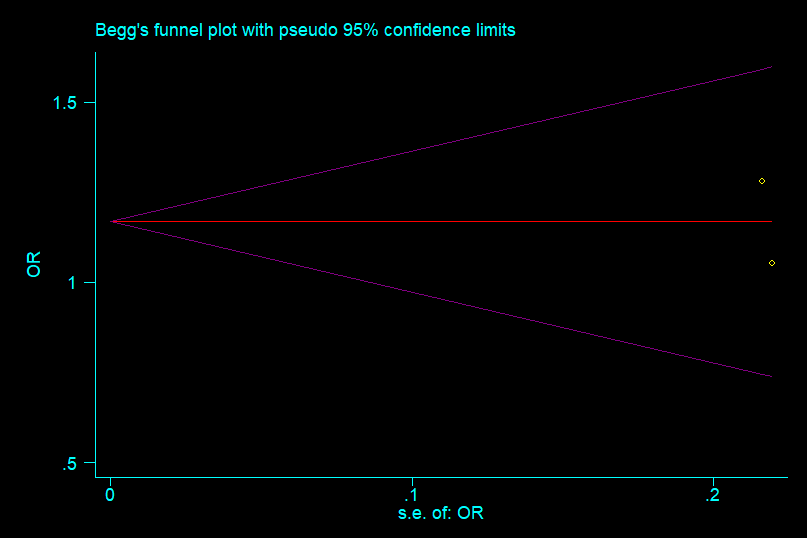


**The Begg funnel plot of LPL rs12678919.** The vertical axis represents OR and the horizontal axis means the standard error (SE) of OR. Horizontal line and sloping lines in the funnel plot represent random effect summary OR and expected 95%CI for a given SE, respectively. Each circle area represents the contribution of each study to the pooled OR


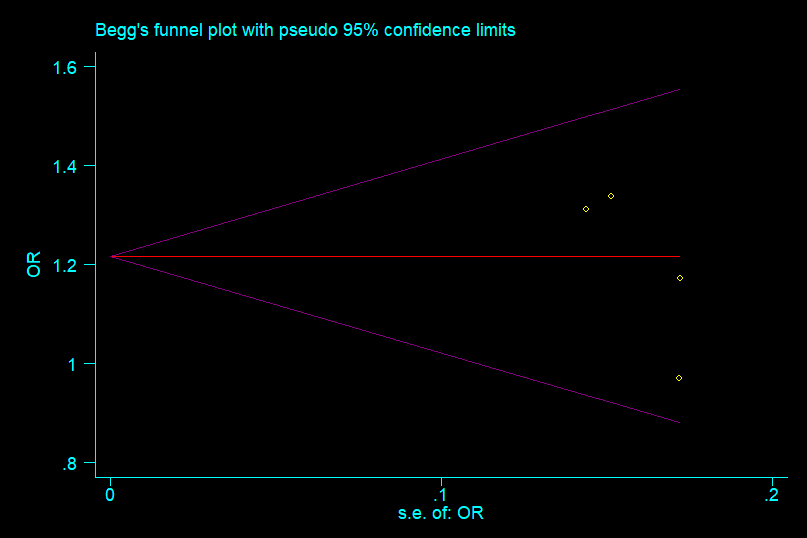


**The Begg funnel plot of ABCG1 rs57137919.** The vertical axis represents OR and the horizontal axis means the standard error (SE) of OR. Horizontal line and sloping lines in the funnel plot represent random effect summary OR and expected 95%CI for a given SE, respectively. Each circle area represents the contribution of each study to the pooled OR


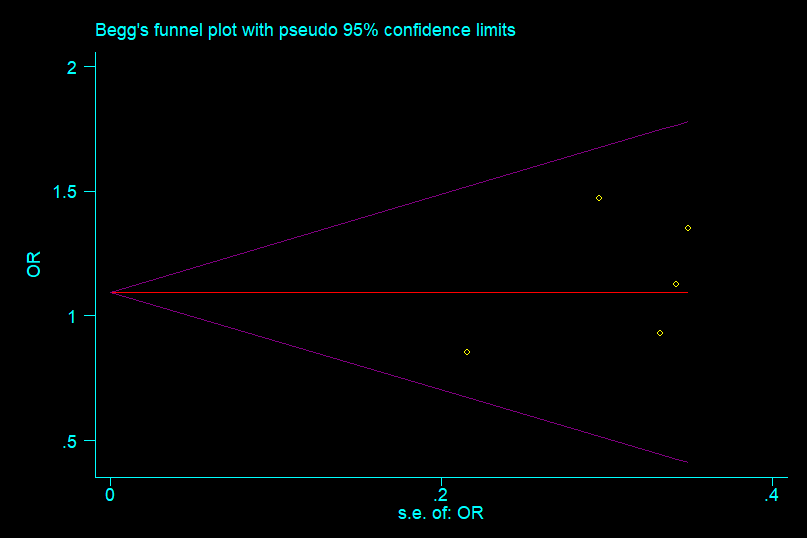


**The Begg funnel plot of CEPT rs2303790.** The vertical axis represents OR and the horizontal axis means the standard error (SE) of OR. Horizontal line and sloping lines in the funnel plot represent random effect summary OR and expected 95%CI for a given SE, respectively. Each circle area represents the contribution of each study to the pooled OR
